# Fluid supplementation accelerates epithelial repair during chemical colitis

**DOI:** 10.1371/journal.pone.0215387

**Published:** 2019-04-19

**Authors:** Juan F. Burgueño, Jessica K. Lang, Ana M. Santander, Irina Fernández, Ester Fernández, Julia Zaias, Maria T. Abreu

**Affiliations:** 1 Division of Gastroenterology, Department of Medicine, University of Miami–Leonard Miller School of Medicine, Miami, FL, United States of America; 2 Department of Immunology and Infectious Diseases, Harvard T. H. Chan School of Public Health, Boston, MA, United States of America; 3 Animal Physiology Unit, Department of Cell Biology, Physiology and Immunology, Universitat Autònoma de Barcelona, Bellaterra, Barcelona, Spain; 4 Division of Veterinary Resources, University of Miami Miller School of Medicine, Miami, FL, United States of America; 5 Department of Pathology and Laboratory Medicine, University of Miami–Leonard Miller School of Medicine, Miami, FL, United States of America; Indiana University School of Medicine, UNITED STATES

## Abstract

The dextran sulfate sodium (DSS) model of colitis is a common animal model of inflammatory bowel disease that causes pain and distress. In this study, we aimed to determine whether fluid supplementation can be used as a welfare-based intervention to minimize animal suffering. C57Bl/6 females undergoing acute colitis by administration of 3% DSS in drinking water were supplemented with 1 mL intraperitoneal injections of NaCl and compared to non-supplemented control mice. Mouse behavior and locomotive activity were assessed on days 5–6 after DSS initiation by means of tail suspension, novel object recognition and open field activity tests. Mice were euthanized after either the acute (day 7) or the recovery phase (day 12) of colitis and inflammation, epithelial proliferation, and differentiation were assessed by means of histology, immunohistochemistry, quantitative PCR, and western blot. We found that fluid-supplemented mice had reduced signs of colitis with no alterations in behavior or locomotive activity. Furthermore, we observed an accelerated epithelial repair response after fluid hydration during the acute phase of colitis, characterized by increased crypt proliferation, activation of ERK1/2, and modulation of TGF-β1 expression. Consistent with these findings, fluid-supplemented mice had increased numbers of goblet cells, upregulated expression of differentiation markers for absorptive enterocytes, and reduced inflammation during the recovery phase. Our results show that fluid hydration does not reduce stress in DSS-treated mice but alters colitis evolution by reducing clinical signs and accelerating epithelial repair. These results argue against the routine use of fluid supplementation in DSS-treated mice.

## Introduction

Inflammatory bowel disease (IBD) refers to a group of pathologies of increasing incidence worldwide that affects more than 1.3 million patients in the United States [1, 2]. The most common forms of IBD, Crohn’s disease (CD) and ulcerative colitis (UC), are characterized by chronic and relapsing inflammation of the gut. Although therapy has greatly improved, the pathogenesis of IBD is still not fully understood, making research in animal models necessary to generate mechanistic insights of these diseases. There are several animal models of IBD. Although none of them recapitulate all pathogenic and clinical features of IBD, they collectively contribute to strengthening our understanding of the processes driving gut inflammation [3, 4].

One of the most common animal models of IBD is chemically-induced colitis by administration of dextran sulfate sodium (DSS). DSS is a sulfated polysaccharide that decreases the thickness of the mucus layer and increases the permeability of the epithelial barrier, facilitating microbial penetration into the mucosa [5, 6]. It is also generally believed that DSS exerts toxic effects in intestinal epithelial cells (IECs), although such effects have not been clearly documented. When administered at 2–5% concentration in drinking water for 5–7 days, the 40 kDa form of DSS causes inflammation in the mid-distal colon that is characterized by mononuclear infiltration of the mucosa, edema, and crypt destruction with formation of erosions and ulcers [7, 8]. This model is simple, rapid, and can be used in genetically modified mice to interrogate the participation of specific proteins in the onset of inflammation.

Given the increasing awareness of animal welfare, it is necessary to attempt to ensure the humane care of the animals used for research and education purposes. Following regulations, animal welfare committees oversee the programs established within each institution to take proper care of animals in research. These committees are increasingly stringent with respect to minimizing any potential animal suffering. One of the main issues with animal models of colitis is the concern that animal welfare is compromised by the colitis itself. Colitis is manifested by diarrhea, rectal bleeding, and weight loss, but more severe forms can further cause dehydration, anemia, and eventually the death of the animal. Furthermore, DSS colitis has been shown to induce hyperalgesia, causing additional distress [9]. To minimize potential animal suffering during DSS-induced colitis, animal welfare committees may require investigators to provide fluid supplementation to mice losing a certain amount of weight.

In the current study, we aimed to determine whether providing supplemental fluid to animals undergoing a standard DSS protocol modifies the course of colitis and affects animal welfare. We hypothesized that the additional fluid would make the weight changes invalid in tracking colitis severity and would reduce animal distress. To our surprise, providing additional fluid did not change inflammation or stress as defined by behavioral alterations, but reduced the signs of colitis and completely modified the epithelial recovery program. Our study demonstrates that investigators should be wary of procedures suggested to improve animal welfare, such as fluid supplementation, as the phenotype of the underlying disease model can be profoundly altered.

## Materials and methods

### Animals

Ten to twelve-week old C57Bl/6 female mice were purchased from Jackson Laboratories (Bar Harbor, USA) and housed in specific pathogen-free conditions, under a controlled temperature (20±2°C) and photoperiod (12h/12h light-dark cycle), with free access to food and water. All cages contained Nestlets (Ancare) for environmental enrichment and were replaced once every 2 weeks. All animal procedures were approved by the Institutional Animal Care and Use Committee (IACUC) at the University of Miami (protocol number 15–223). The University of Miami is an Association for Assessment and Accreditation of Laboratory Animal Care (AAALAC) International-accredited facility.

### Experimental design and colitis assessment

For each experiment, 3 to 5 mice were randomly distributed in standard shoebox cages and allowed to adapt for at least 5 days before colitis induction. Colitis was induced by addition of 3% DSS (40–50 kDa; Affymetrix/USB, ThermoFisher Scientific) to drinking water for 6 consecutive days. DSS solution was freshly prepared and replaced every other day. Animals were assessed daily for individual weight loss, stool consistency, and fecal blood to build up a disease activity index (DAI) to follow up colitis, as previously described [8]. Briefly, weight loss percentage ranged from 0 (no weight loss) to 4 (>15% loss) in increments of 5%; the stool consistency scale ranged from 0 (normal stool consistency) to 4 (marked diarrhea); and blood in stool ranged from 0 (no blood as determined by Hemoccult test (VWR)) to 4 (gross, apparent blood in stool). Values for these parameters were averaged to obtain a daily DAI score for each mouse. Mice were immediately euthanized when they met one of the following endpoint criteria: losing more than 30% of the initial body weight or having a combined DAI score averaging >3.5 points. No animals died or met humane endpoint criteria prior to the end of the study.

**Fig 1:**
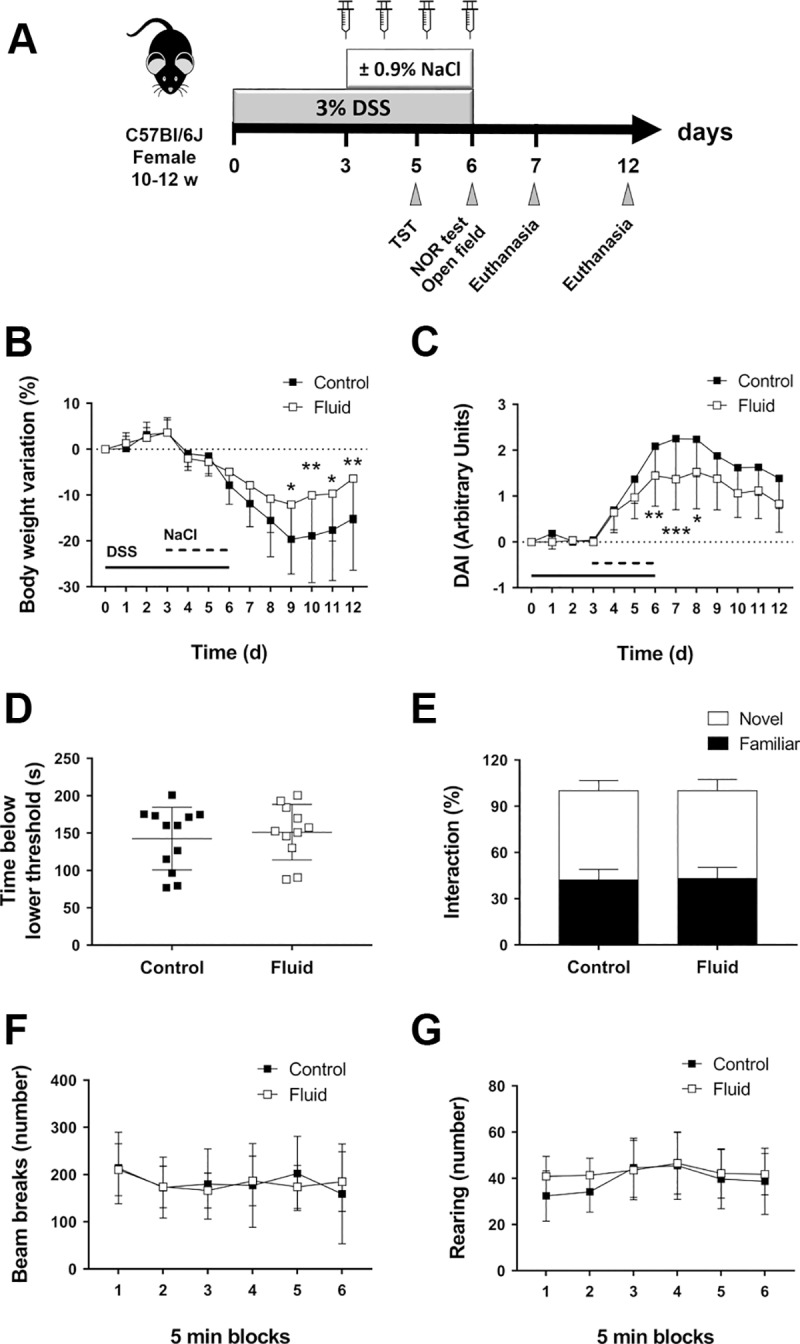
Fluid supplementation ameliorates signs of colitis without altering mouse behavior. **A)** Diagram showing the procedures that mice underwent throughout the whole study. One mL of 0.9% NaCl solution was injected IP in fluid-supplemented mice from days 3 to 6 after beginning of DSS administration. **B)** Body weight variation (n = 16–17 until day 7, n = 9 from days 7–12; *P<0.05 and **P<0.01, as determined by two-way ANOVA). Data represent values for 2 different studies, which explains the high variability. ANOVA detected “Fluid” as a factor with a very significant source of variation (*P*<0.0001). DSS administration days are represented with a continuous line; fluid administration days are represented with a dashed line. **C)** DAI (n = 16–17 until day 7, n = 9 from days 7–12; *P<0.05, **P<0.01, and **P<0.001, as determined by two-way ANOVA). “Fluid” was detected as a very significant factor of variation (*P*<0.0001). DSS administration days are represented with a continuous line; fluid administration days are represented with a dashed line. **D)** Tail suspension test (TST; n = 11–12; two-tailed t-test). **E)** Novel object recognition test (NOR; n = 11–12; two-tailed T-test comparing interactions with novel object). **F)** Open field activity test (n = 11–12; two-way ANOVA). **G)** Rearing behavior (n = 11–12; two-way ANOVA).

Mice undergoing fluid supplementation received daily intraperitoneal (IP) injections with 1 mL of 0.9% NaCl (B. Braun Medical Inc.) on days 3 to 6 after DSS initiation (Fig 1A). In order to control for stress and handling effect, untreated mice were equally manipulated by inserting a needle. The entire experiment was repeated twice. In the first set of experiments, in order to ascertain if fluid supplementation changes DSS intake, all mice from the same cage received the same treatment and DSS consumption was recorded daily (n = 3 cages per experimental group; S1A Fig). Given that DSS consumption was not altered by fluid supplementation, during the second set of experiments mice supplemented with IP fluid were cohoused with non-supplemented mice to control for “cage” effects.

### Behavioral assessment

The tail suspension test (TST) was performed on day 5 to evaluate depressive behavior [10]. Control and fluid-supplemented mice were simultaneously suspended for 6 minutes from a vertical metal bar attached to a strain gauge set to detect animal movement (Med Associates Inc). The time spent by the mice below a preset threshold during the last 4 minutes was calculated to determine the duration of immobility.

The novel object recognition (NOR) test was performed on day 6 to assess cognitive behavior, working memory, and preference for novelty [11, 12]. Briefly, the mice were individually placed into a rectangular, Plexiglass open field of 30 x 19 x 19 cm (NOR chamber), with two identical objects positioned on the extremes of the field and separated approximately 5 cm from the walls. Mice were allowed to explore the two objects for 10 min and were then transferred to an empty open field for 10 additional minutes. After this time, mice were transferred again to the NOR chamber and re-exposed to the same object of the first phase, together with a novel object. Mice were allowed to explore the arena for 5 minutes and the time spent interacting with each object was filmed from above and measured. The position of each object was alternated between trials to avoid any misinterpretation of data.

The open field activity test was performed on day 6 to assess locomotor activity. Fluid-treated or control mice were individually placed in an open field arena (43.2 x 43.2 x 30.5 cm) fitted with 16 evenly spaced infrared sources and sensors juxtaposed around the periphery of the chamber (Med Associates). Beam breaks (50 ms sampling rate) were recorded for 30 minutes in a computer attached to the chambers. Three consecutive beam breaks were considered an episode of horizontal activity. The number of beam breaks and times the mice stood on their hind legs (rearing) in each one of the 5-minute blocks were calculated.

All tests were performed in the presence of a white noise generator set at 55 dB to cover intermittent environmental sounds.

### Euthanasia and tissue collection

Mice were euthanized on days 5, 7, or 12 to address the effects of fluid supplementation during acute and recovery phases of colitis. Four hours before euthanasia, mice were orally gavaged with fluorescein isothiocyanate-dextran (3–5 kDa, FD4; Sigma Aldrich) for *in vivo* permeability determination. Euthanasia was performed by cardiac puncture exsanguination under isoflurane (Piramal Critical Care) anesthesia, followed by cervical dislocation. Blood was collected in heparinized syringes and centrifuged to obtain plasma, which was subsequently used for FD4 quantification in plasma, acute phase protein determinations, and protein electrophoresis. Colons were removed from the abdominal cavity, flushed with ice-cold Hank’s Balanced Salt Solution (HBSS), measured from the cecocolic junction to the anus, and cut into 4 longitudinal pieces. The first piece was mounted as a Swiss roll and fixed in 4% paraformaldehyde for histology; the second piece was placed in RNA*later* stabilization solution (ThermoFisher Scientific) for RNA extraction; and the third and fourth pieces were snap-frozen for western blot and myeloperoxidase (MPO) determinations.

### In vivo permeability determination

Epithelial barrier function *in vivo* was assessed by the leakage of orally gavaged FD4 into blood. Mice were given 600 mg/kg FD4 four hours prior to euthanasia and fluorescence in plasma at 490/525 nm was measured in a CLARIOstar Reader (BMG LABTECH). Concentration of FD4 in plasma was extrapolated by generating a standard curve, as previously described [13].

### Biochemical assessments of inflammation

Determination of the acute phase protein serum amyloid A (SAA) in plasma was performed by means of a mouse SAA/SAA1 PicoKine ELISA kit (Boster), following manufacturer’s instructions. Plasma protein concentration was assessed by electrophoresis in a Helena SPIFE 3000 device with the use of Split Beta gels (Helena Laboratories Inc). Electrophoretic analyses were conducted by the Pathology Research Resources Laboratory (Division of Comparative Pathology, University of Miami).

### Myeloperoxidase activity

Snap-frozen longitudinal sections of colon were homogenized by means of a BeadBlaster 24 (Benchmark Scientific) in a 13.7 mM solution of hexadecyltrimethylammonium bromide (HTAB; Sigma Aldrich) in 50 mM phosphate buffer. Aliquots of the supernatants were tested for their ability to oxidize *o*-dianisidine (Sigma Aldrich) in the presence of hydrogen peroxide and compared to the activity of different MPO standards run in parallel. Resulting MPO activity was corrected to the initial weigh of each sample.

### Histologic score and morphometric measurements

Histologic assessment of colitis was performed by an investigator blinded to the study design. Hematoxylin & eosin sections from mid-distal colon of each animal were evaluated as previously described for chemical induced colitis [14]. Ten different areas were evaluated for inflammation severity and crypt damage and averaged to obtain a histologic score.

For epithelial preservation analysis, micrographs from each colon roll were taken at a low magnification (20X) in a BZ-X700 microscope (Keyence). Depending on crypt damage, the surface of the epithelium along the total length of each sample was measured and classified by an observer blinded to the study design into: normal epithelium, partial crypt loss (less than 2/3 of crypt persistent), epithelial lining (total crypt loss with a superficial epithelial layer covering the wounded area), total loss, or hyperproliferative areas (hyperplastic crypts with hyperchromatic staining and high nuclear to cytoplasmic ratio). Measurements were performed by means of the ImageJ software (National Institutes of Health). Results are expressed as the percentage of the total length of the colon sample.

For crypt morphometry, the height of at least 15 well-oriented crypts from different areas was determined by means of the ImageJ software (National Institutes of Health).

For goblet cell counting, slides were stained for 30 min in a 1% Alcian blue (8GX, Sigma Aldrich) solution prepared in 3% acetic acid pH 2.5, and subsequently counterstained for 5 min with 0.1% nuclear fast red solution (Vector Labs). Ten to twenty-five well-oriented crypts from different areas were counted by means of a manual cell counter plugin in ImageJ (NIH).

### Immunohistochemistry

Deparaffinized tissues were treated for antigen retrieval with boiling citric acid for 15 min. Endogenous peroxidases were neutralized by incubation in 0.3% hydrogen peroxide in distilled water for 30 min, and unspecific binding was blocked with 5% donkey serum in PBS + 0.5% Tween-20 for 1 hour. Slides were then incubated overnight at 4°C with primary antibodies for Ki67 (ab16667, Abcam; 1/500) and p-ERK1/2 (AF1018, R&D systems; 5 μg/mL), followed by addition of biotin-conjugated secondary antibody (B21078, ThermoFisher Scientific; 1/200) for 1 hour at room temperature, enhancement with Vectastain ABC kit (Vector Laboratories Inc) for 30 minutes, and development with the ImmPACT DAB kit (Vector Laboratories Inc). Tissues were counterstained with Mayer’s hemalum solution (EMD Millipore) and analyzed under a BZ-X700 microscope (Keyence). For Ki67 quantification, the number of Ki67^+^ nuclei were quantified and normalized to the total number of nuclei in 10–20 well-oriented crypts.

### Quantitative PCR analysis

Colon samples embedded in RNA*later* solution were homogenized in RNA-bee (Tel-Test Inc) and RNA was isolated using phenol-chloroform extraction [15], followed by an additional precipitation step in LiCl to avoid polymerase inhibition by residual DSS [16]. 500 ng of RNA were retro-transcribed by using the PrimeScript RT reagent Kit (Takara Bio Inc) and amplified by means of the SYBR Premix Ex Taq (Takara) on a LightCycler 480 II instrument (Roche Applied Science). A list of the primers used can be found in S1 Table. Absence of coamplification products was assured by generating a final melting curve for each reaction. mRNA level of expression of the genes of interest was normalized to the housekeeping gene β-actin and calculated by means of the ΔΔCt method [17].

### Western blot

Protein from colon longitudinal strips was isolated by homogenization in RIPA lysis buffer supplemented with Halt Protease and Phosphatase Inhibitor Cocktail (ThermoFisher Scientific) and quantified by BCA assay (ThermoFisher Scientific). 15 μg of protein were separated in NuPAGE 4–12% Bis-Tris gels and transferred to a nitrocellulose membrane with the iBlot2 Dry Blotting System (ThermoFisher Scientific). Membranes blocked in 5% non-fat dry milk underwent overnight incubation at 4°C with primary antibodies for phospho-ERK1/2 (4370, Cell Signaling Technology; 1/2000) and total ERK1/2 (9102, Cell Signaling Technology; 1/1000) diluted in 5% bovine serum albumin (Sigma Aldrich) in Tris buffered saline + 0.5% Tween-20 (TBST). Detection was performed with horseradish peroxidase-conjugated anti-rabbit antibody (G-21234, ThermoFisher Scientific; 1/10,000). Membranes were developed with Supersignal West Dura chemiluminescent substrate (ThermoFisher Scientific) and visualized on a myECL Imager (ThermoFisher Scientific). HRP-conjugated mouse antibody to β-actin (A3854, Sigma Aldrich, 1/20,000) was used to certify equal loading of samples.

### Statistical analysis

Results are presented as mean values and standard deviation (SD). All data were compared using one-way or two-way ANOVA, followed by Sidak’s post-hoc test (unless otherwise stated). Data analysis and plot were performed with GraphPad Prism 7.0 software (GraphPad Software Inc). A *P* value <0.05 was considered to be significant.

## Results

### Fluid supplementation improves signs of experimental colitis

DSS-induced colitis causes mild to severe bloody diarrhea, which in turn may lead to weight loss, dehydration, and anemia [8, 18]. To determine the utility of fluid supplementation in managing the clinical signs of colitis and improving overall condition and welfare, mice receiving IP fluid injection during acute DSS-induced colitis were compared to non-supplemented control mice. Fluid injection altered neither water consumption (S1A Fig) nor body weight of mice during the supplementation period (days 3 to 6 after DSS addition), but significantly improved body weight loss from days 9 to 12 (Fig 1B). In addition to changing weight, fluid supplementation also altered the consistency and the presence of blood in stool. Fluid supplementation significantly ameliorated rectal bleeding and stool consistency during the peak of colitis, as demonstrated by decreased DAI (days 5 to 8, Fig 1C and S1B and S1C Fig). Overall, fluid treatment significantly improved the course of DSS colitis in terms of body weight loss and DAI, as indicated by two-way ANOVA analysis (*P*<0.0001 for “Fluid” factor in both parameters), highlighting the usefulness of this intervention to improve welfare.

To address whether mice were experiencing dehydration during colitis, body condition and skin hydration were evaluated by a veterinarian as in [18] throughout the experiment. We additionally measured total protein in plasma, which increases during dehydration [19]. The body condition of mice was normal throughout the entire experiment (not shown) and we did not see any significant increases in total plasma protein in either the fluid-supplemented or non-supplemented mice (S1D Fig), suggesting that moderate colitis does not induce overt dehydration. However, we observed a significant reduction in plasma proteins of control mice on day 12, suggesting that these mice may have a protein-losing enteropathy as occurs in colitis (S1D Fig). Taken together, these data demonstrate that fluid supplementation can alter the overall DAI, a critical readout in most experiments, even in the absence of dehydration.

### Fluid supplementation does not alter mouse behavior during DSS colitis

Acute colitis by DSS impairs cognitive [20] and locomotive behavior [21], and has been shown to induce hyperalgesia [9, 22], which can further modify mouse behavior. To determine whether fluid supplementation reduces mouse stress during acute colitis, DSS-treated control and fluid-supplemented mice underwent the TST, NOR, and open field activity tests on the indicated days (Fig 1A). Our data show that fluid supplementation did not change the immobility time spent below the lower threshold in the TST (Fig 1D), the percentage of interactions with novel and familiar objects in the NOR test (Fig 1E), or the locomotive (Fig 1F) or the rearing behavior of the mice in the open field test (Fig 1G). Moreover, anxiety-like behavior, as defined by the number of beam breaks in the central and peripheral areas of the open field, was not modified by fluid supplementation (S1E and S1F Fig). Taken together, our findings show that fluid supplementation during DSS administration improves the clinical signs of colitis but does not affect signs of stress.

### Fluid supplementation does not ameliorate inflammation

**Fig 2:**
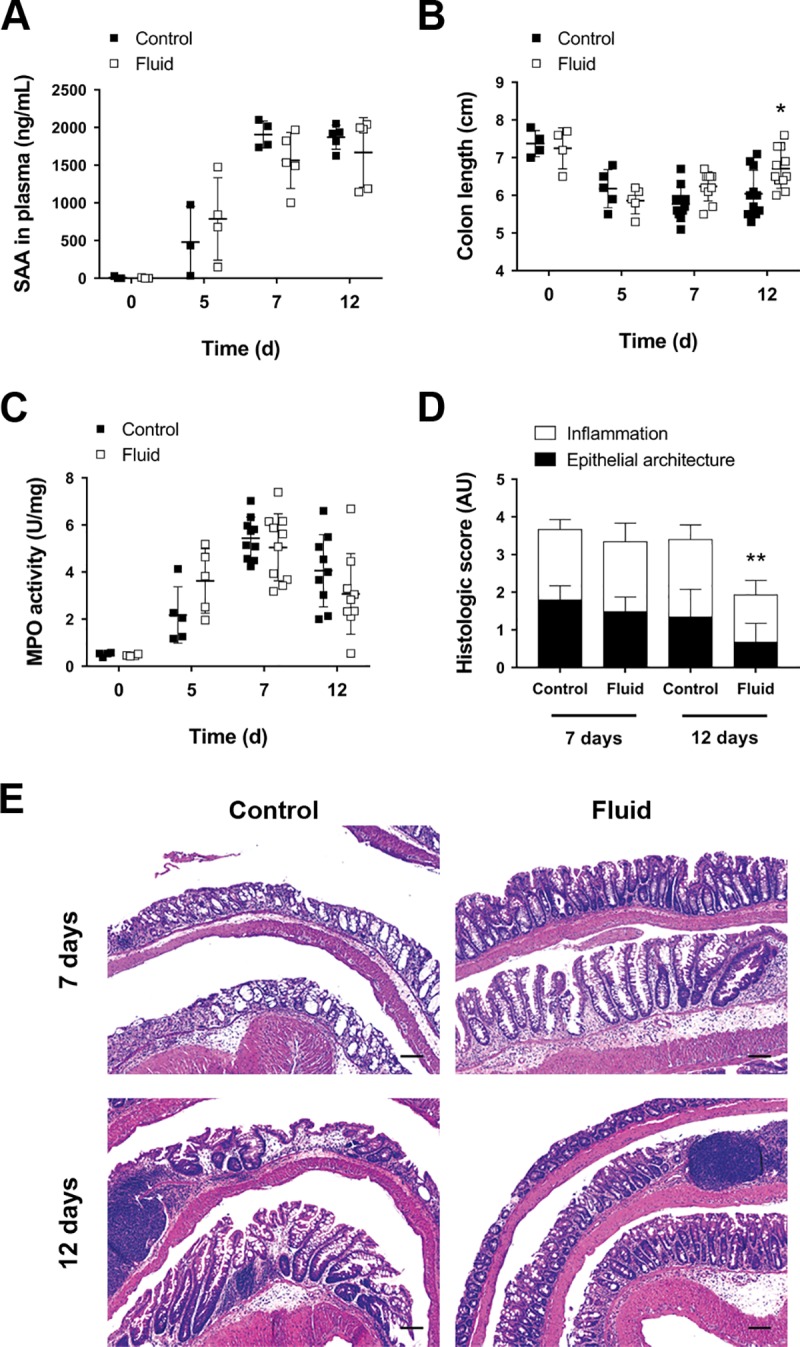
Fluid supplementation does not reduce severity of colitis. **A)** Serum amyloid A in plasma (SAA; n = 3–5; two-way ANOVA). **B)** Colon shortening during colitis (n = 4–11; *P<0.05 for fluid vs control on day 12, as determined by two-way ANOVA). **C)** Myeloperoxidase activity in colon homogenates (MPO; n = 4–10; two-way ANOVA). **D)** Histologic score divided in inflammation and epithelial architecture scores (n = 7–9; **P<0.01 for fluid vs control on day 12, as determined by two-way ANOVA). **E)** Representative H&E micrographs showing colon mucosa on days 7 and 12. Scale bars = 200 μm.

Since amelioration of clinical signs is associated with reduced colitis severity, we next sought to determine the effect of fluid administration on inflammation. To evaluate systemic inflammation, we measured the concentration in plasma of the acute phase protein SAA, which has been identified as a very sensitive marker correlating with disease activity during colitis [23, 24]. SAA progressively increased in plasma during colitis development, peaking 7 days after the beginning of DSS administration (Fig 2A). However, no differences were observed between fluid-supplemented and control mice, suggesting that DSS colitis causes systemic manifestations of inflammation, but these are not affected by fluid supplementation.

We next focused on the effects of fluid supplementation on colonic inflammation. Colon inflammation was assessed by colon shortening (Fig 2B), increased neutrophil infiltration (as determined by MPO activity, Fig 2C), and upregulation of the pro-inflammatory cytokines IL-1β and TNF (S2A and S2B Fig). Although we did not observe significant differences between fluid supplementation and control groups in MPO activity and cytokine expression, a significant increase in colon length was seen in fluid-supplemented mice on day 12 (Control = 6.04 cm (SD 0.62) vs Fluid = 6.71 cm (SD 0.52), *P*<0.05; Fig 2B), suggesting an accelerated recovery from inflammation. Histologic analysis corroborated a reduction in the extent of the inflammatory infiltrate observed in fluid-treated mice on day 12, as well as an improved epithelial architecture, with fewer erosion areas (Control = 3.4 AU (SD 1.03) vs Fluid = 1.93 (SD 0.84), *P*<0.001; Fig 2D and 2E). These data suggest that fluid supplementation during colitis does not reduce systemic or local acute inflammation but may accelerate the recovery phase.

### Fluid supplementation promotes epithelial proliferation and regeneration during colitis

**Fig 3:**
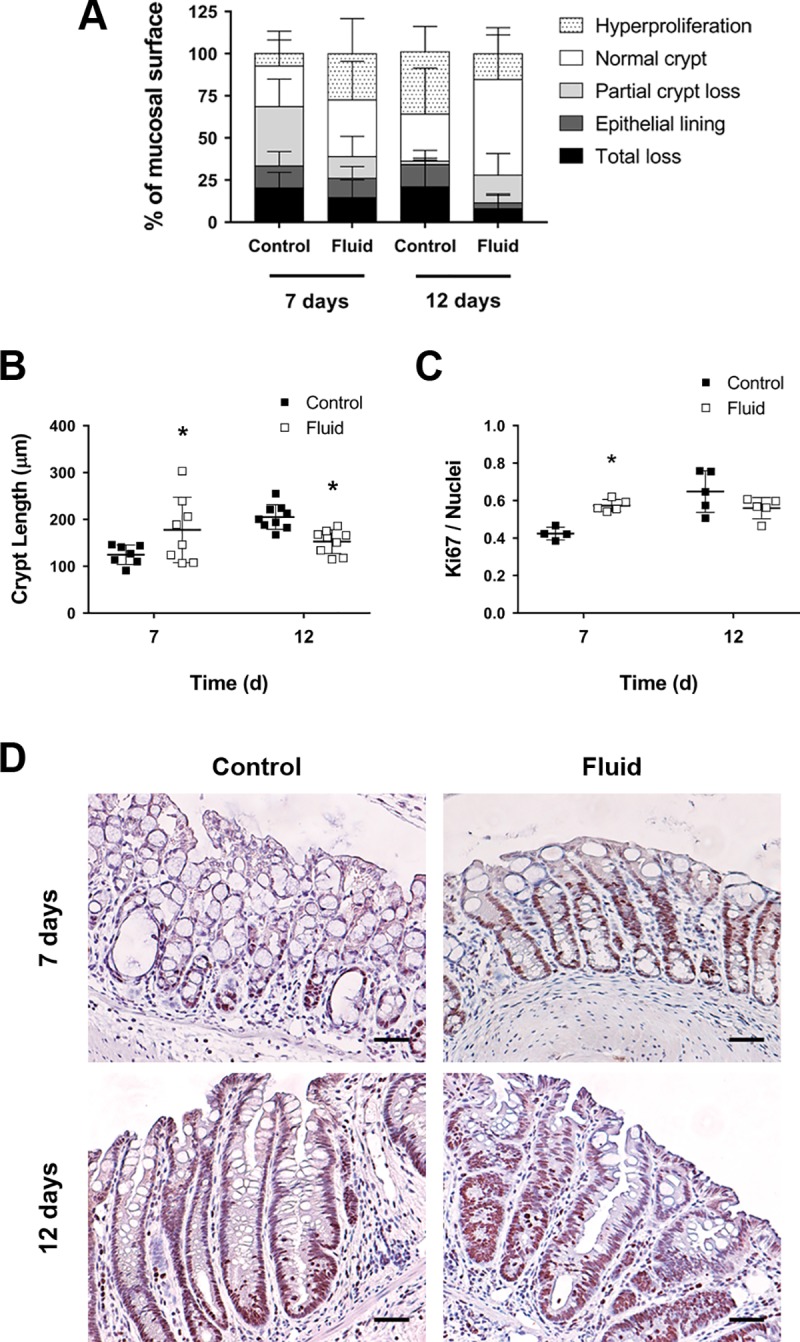
Fluid resuscitation increases epithelial proliferation. **A)** Quantification of the mucosal surface covered by normal, damaged, and hyperproliferative epithelium (n = 7–9; *P*<0.05 for fluid vs control on day 12 for “normal crypt” and *P*<0.05 for fluid vs control on days 7 and 12 for “hyperproliferation”, as determined by two-way ANOVA). ANOVA detected a significant interaction effect between “Fluid” and “Time” factors (*P*<0.001). **B)** Crypt length (n = 7–9; *P<0.05 for fluid vs control on days 7 and 12, as determined by two-way ANOVA). ANOVA detected a significant interaction effect between “Fluid” and “Time” factors (*P*<0.001). **C)** Ratio of Ki67 positive cells per total number of epithelial nuclei (n = 4–5; *P<0.05 for fluid vs control on day 7). ANOVA detected a significant interaction effect between “Fluid” and “Time” factors (*P*<0.01). **D)** Representative micrographs showing Ki67 positive cells in colon crypts. Scale bars = 200 μm.

Given the results of our assessment of inflammation, we next asked whether fluid supplementation changed epithelial repair. To address this question, we performed morphometric assessments of the epithelium of inflamed mice. Mucosal areas with different epithelial architecture were measured and classified by a pathologist (JZ) to assess the degree of preservation and regeneration of the epithelium. DSS-treated control mice during acute colitis (day 7) were characterized by extensive areas of severe epithelial cell loss. In contrast, fluid-supplemented mice showed a significant increase in the proportion of mucosal surface covered by hyperproliferative crypts (Control = 7.4% (SD 13.3) vs Fluid = 27.5% (SD 20.9), *P*<0.05; Fig 3A). Additionally, the proportion of hyperproliferative crypts was greater in DSS control mice in the recovery phase (12 days), whereas fluid-supplemented animals had a higher percentage of mucosa covered by normal crypts (Control = 27.9% (SD 27.3) vs Fluid = 56.7% (SD 30.8), *P*<0.05; Fig 3A). In agreement with these findings, we found that fluid-supplemented mice had significantly taller crypts during the acute phase and a return to normal height crypts during the recovery phase of colitis (Fig 3B). Of note, the ANOVA revealed a strong significance for the interaction between “Time” and “Fluid” factors for regenerative epithelium and crypt length (*P*<0.001 for both). This significant interaction implies that fluid supplementation had opposite effects on days 7 and 12, strongly suggesting that fluid accelerates epithelial regeneration by enhancing proliferation during the early phase of colitis (day 7).

Given our findings with respect to crypt height in response to fluid resuscitation, we next asked whether fluid supplementation changes proliferation in the recovering epithelium. To address this question, we analyzed the expression of the proliferation marker Ki67 by immunohistochemistry. Consistently, we observed that Ki67+ nuclei were significantly increased in fluid-supplemented mice when compared to untreated controls on day 7 after DSS administration (Fig 3C and 3D, upper panels). These findings were further corroborated by quantitative analysis of the expression of *Ki67* and *Axin-2* transcripts (S3A and S3B Fig): overall, the ANOVA test detected a significant effect for fluid supplementation in upregulating proliferative-associated transcripts (*P*<0.001 for *Ki67*, and *P*<0.05 for *Axin-2*) along the experimental time-lapse. Taken together, our data indicate that fluid supplementation enhances mucosal wound healing through increased epithelial proliferation.

### Fluid resuscitation accelerates epithelial differentiation in the recovery phase of colitis

**Fig 4:**
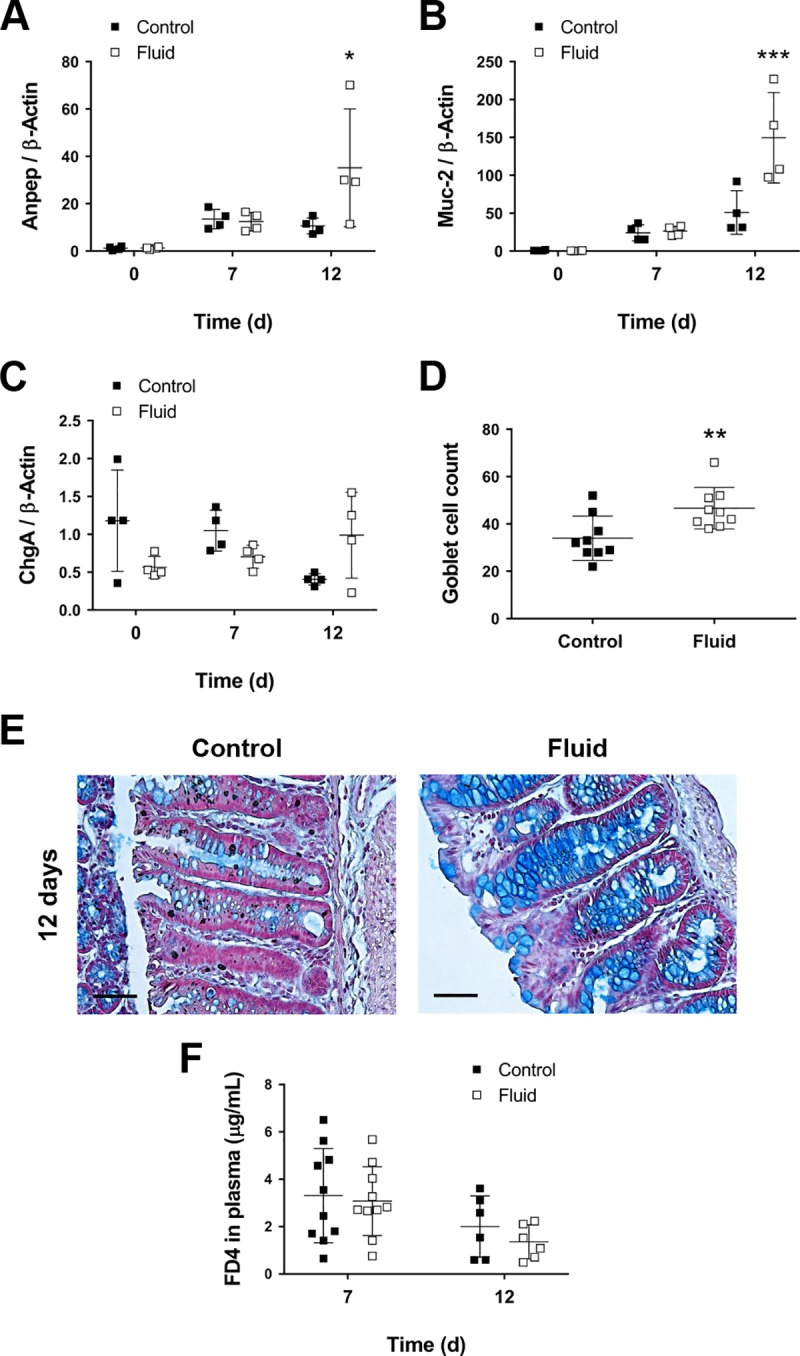
Epithelial differentiation is accelerated in fluid-supplemented mice. **A)** Anpep mRNA expression levels (n = 4; *P<0.05 for fluid vs control on day 12, as determined by two-way ANOVA). **B)** Muc-2 mRNA expression levels (n = 4; **P<0.01 for fluid vs control on day 12, as determined by two-way ANOVA). **C)** ChgA mRNA expression levels (n = 4). **D)** Goblet cell count per crypt on day 12 (n = 9; **P<0.01 for fluid vs control, as determined by two-tailed t-test). **E)** Representative micrographs showing increased number and maturation of Goblet cells in the crypts of fluid-treated mice. Scale bars = 50 μm. **F)** *In vivo* permeability assay as determined by the presence of orally gavaged FD4 in plasma (n = 6–10). ANOVA detected a significant difference in “Time” factor (*P*<0.05).

Next, we sought to evaluate the effects of fluid therapy on cell differentiation and epithelial barrier function as another dimension in the process of wound healing. Differentiation of the proliferative IECs was addressed by determining the expression of transcripts for absorptive (alanine aminopeptidase; *Anpep*), secretory (Mucin-2; *Muc-2*) and enteroendocrine (chromogranin A; *ChgA*) lineages. We had previously observed a higher proportion of the epithelial layer with a normal morphology in fluid-treated mice during the recovery phase of colitis (Figs 2E and 3A). Consistently, we found significantly increased levels of mRNA for *Anpep* and *Muc-2* (Fig 4A and 4B) in fluid-supplemented mice on day 12, indicating that differentiation into absorptive enterocytes and mucus-secreting goblet cells occurs earlier upon fluid supplementation. Conversely, we did not find differences in the expression of the enteroendocrine marker ChgA (Fig 4C). We further assessed differentiation into goblet cells by staining crypts with Alcian blue, which is used to visualize acidic mucopolysaccharides. DSS control mice had longer crypts with fewer mucus-producing goblet cells (Control = 34 cells (SD 9.35) vs Fluid = 46.7 cells (SD 8.75, *P*<0.01; Fig 4D and 4E), corroborating that the differentiation of the hyperproliferative crypts is advanced in time by fluid injection.

To test the recovery of the epithelial barrier function, we orally administered mice 4 kDa FITC-dextran (FD4) four hours before euthanasia and measured FITC fluorescence in plasma. Luminal FD4 can leak through ulcers, erosions, and leaky epithelial junctions to the blood, where it can be quantified by fluorescence reading. Our results show that the presence of FD4 in plasma was significantly decreased during the recovery phase of colitis (12 days) as compared to the acute phase (7 days) (*P*<0.011 for “Time” factor; Fig 4F). However, fluid supplementation did not improve epithelial permeability. Overall, our results suggest that intraperitoneal fluid administration enhances epithelial restoration by accelerating the proliferation and differentiation at early stages of injury. Permeability by day 12 of recovery is at near normal levels in both control and fluid-supplemented mice.

### Epithelial proliferation and differentiation in fluid-supplemented mice is associated with differential modulation of ERK1/2 activation and TGF-β1 expression

**Fig 5:**
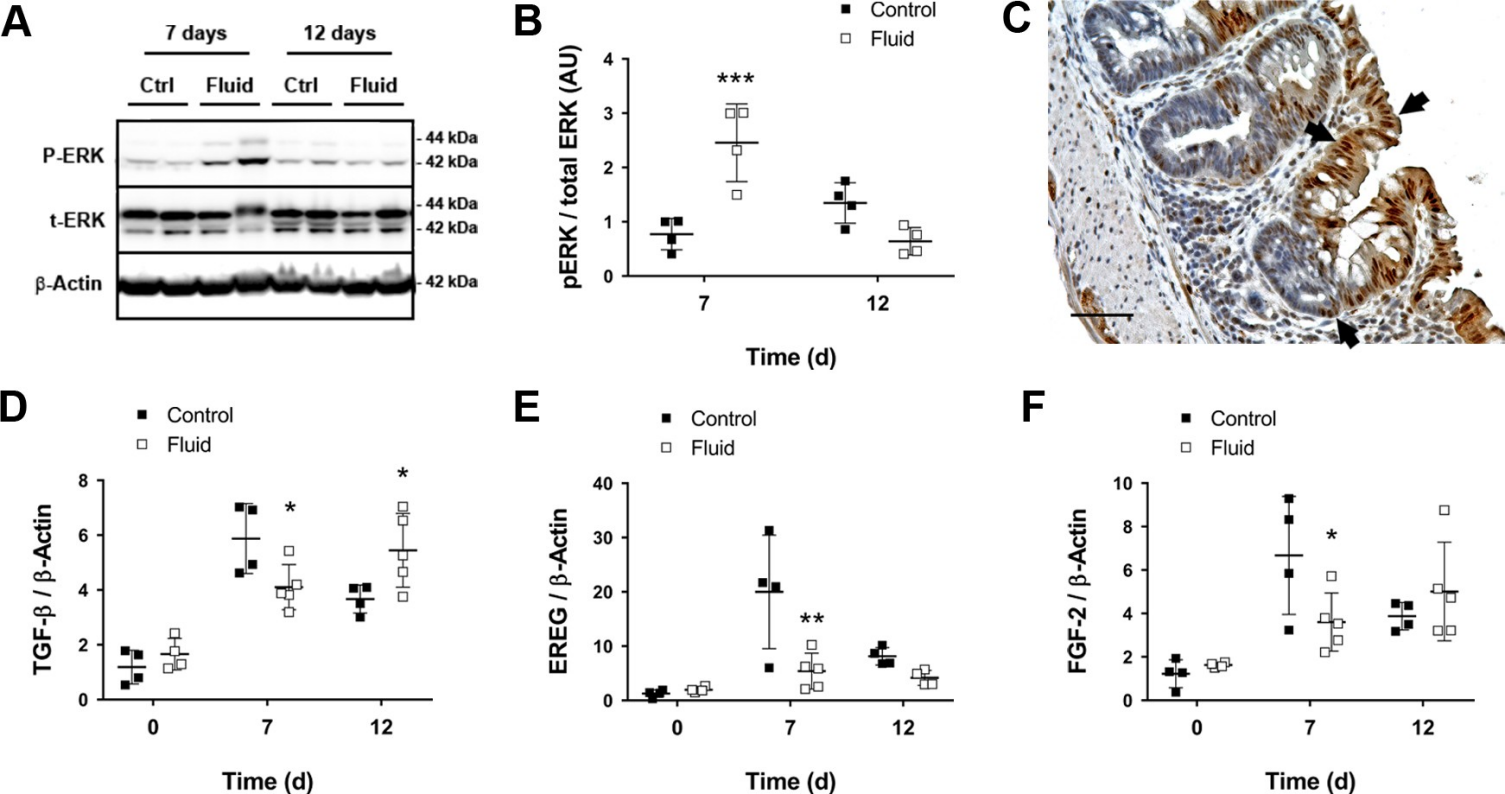
Fluid administration activates ERK1/2 and alters expression of TGF-β to regulate epithelial proliferation and differentiation. **A)** Representative images of phosphorylated and total ERK1/2. **B)** Quantification of the ratio between p-ERK1/2 and t-ERK1/2 (n = 3–4; ***P<0.001 for fluid vs control on day 7, as determined by two-way ANOVA). **C)** High power field micrograph showing nuclear expression of p-ERK1/2 (black arrows). Scale bars = 50 μm. **D)** TGF-β1 mRNA expression levels (n = 4–5; *P<0.05 for fluid vs control on days 7 and 12, as determined by two-way ANOVA). ANOVA detected a significant interaction effect between “Fluid” and “Time” factors (*P*<0.01). **E)** EREG mRNA expression levels (n = 4–5; **P<0.01 for fluid vs control on day 7, as determined by two-way ANOVA). **F)** FGF-2 mRNA expression (n = 4–5; *P<0.05 for fluid vs control on day 7, as determined by two-way ANOVA).

We finally sought to identify the signaling pathways that may be responsible for increased early proliferation and subsequent differentiation in response to fluid supplementation. Several growth factors and signaling cascades promote IEC proliferation [25–27]. Among these, the epidermal growth factor receptor (EGFR) and the downstream mitogen-activated protein kinase (MAPK) pathways are implicated in epithelial proliferation during DSS colitis and wound healing [28, 29]. Therefore, we determined ERK1/2 activation after fluid supplementation in whole colon lysates. Interestingly, phosphorylation of ERK1/2 was increased in fluid-treated mice when compared to untreated controls on day 7, whereas ERK1/2 activation on day 12 was slightly decreased in fluid-supplemented mice (Fig 5A and 5B). Activation of phospho-ERK1/2 occurred preferentially in proliferating and migrating IECs, which displayed intense nuclear staining (Fig 5C, arrows). Additional phospho-ERK1/2 staining could also be observed in immune cells infiltrating the *lamina propria* and neurons in the myenteric plexus. These data point out that the proliferative response mediated by fluid supplementation during colitis occurs via activation of ERK1/2.

To determine the growth factors involved in fluid-elicited epithelial regeneration and differentiation, we examined the expression of different genes associated with epithelial regeneration after injury, including growth factors such as EGF, amphiregulin, epiregulin (EREG), fibroblast growth factor-2 (FGF-2), and transforming growth factor β1 (TGF-β1) [30–33], the prostaglandin E synthase 2 [34], or the cytokines IL-17 and IL-22 [30, 35]. We did not observe significant differences in transcript expression for most of these genes (data not shown). However, we found that fluid supplementation downregulated *TGF-β1* transcript levels on day 7 and upregulated them on day 12 when compared to DSS control mice (Fig 5D). Since TGF-β1 inhibits IEC proliferation and promotes differentiation [36–38], downregulation of this peptide during the acute phase of colitis can facilitate the mitogenic effects of ERK1/2, whereas TGF-β1 upregulation in the recovery phase can enhance differentiation. We also observed significant upregulation of the growth factors EREG and FGF-2 in DSS control mice on day 7 (Fig 5E and 5F). Interestingly, the mRNA levels of *EREG* and *FGF-2* in fluid-treated mice on day 7 were similar to the levels of all mice on day 12, pointing out once again that fluid therapy accelerates the regenerative response. Taken together, these data suggest that fluid-induced epithelial proliferation and subsequent differentiation is driven by the coordinated activation of the MAPK ERK1/2 and modulation of *TGF-β1* expression.

## Discussion

Supportive care interventions can be required by institutional committees to manage welfare-threatening conditions in several animal models of disease. In the chemical model of acute colitis induced by DSS, dehydration and weight loss can be addressed by fluid supplementation of mice. In this study, we demonstrate that IP fluid administration during moderate DSS colitis does not affect stress but leads to earlier recovery from epithelial injury. The improvement in epithelial healing is not associated with an appreciable change in inflammation, but with an increased proliferative response of the crypts that flank the ulcers. Subsequently, accelerated regeneration of the epithelium leads to earlier epithelial differentiation and resolution of erosions and ulcers. These findings have very serious practical implications for researchers to consider in experimental design, as well as for the IBD community.

In a constant effort to ensure humane manipulation and treatment of animals in research protocols, it is the duty of the IACUC committees to require investigators to refine their procedures to minimize animal pain and suffering. The IBD model of DSS colitis is known to induce moderate to severe bloody diarrhea [8, 39], which may cause anemia and dehydration [18]. In this study, we tested whether fluid supplementation can be used as a refining intervention to improve the welfare of mice undergoing DSS colitis. We chose to inject 1 mL of isotonic saline because that is the standard volume commonly used in intraperitoneal administration and is equivalent to half of the maximum volume allowed via this route [40, 41]. To address the welfare of mice, we combined the evaluation of body weight and condition, colitis signs, and indirect parameters to assess dehydration. Our data show that no overt dehydration occurs during moderate colitis, as body condition was minimally affected throughout the experiment and protein levels in plasma were similar to those previously described [42]. However, in all likelihood there are differences in hydration state between mice that are responsible for the improved body weight and DAI observed in fluid-supplemented groups. We further evaluated animal stress by performing behavior tests. It has been described that DSS colitis induces changes in cognitive and locomotive behavior, reducing the exploratory conduct, the time spent in the center of an open field, and locomotion [20–22]. In our hands, amelioration of colitis signs did not alter the behavior of fluid-supplemented mice in any of the tests performed. This unexpected finding may be explained by the fact that systemic and colon inflammation were similar between experimental groups on days 5 to 7, as determined by SAA, MPO, IL-1β, and TNF. IL-1 and TNF can activate the vagus nerve, which innervates the colon, to play important roles in the brain, inducing “sickness behavior” [43–45]. Taken together, our data suggest that fluid supplementation does not affect stress or inflammation but improves disease activity by accelerating epithelial repair in the acute phase of colitis.

Administration of 1 mL of 0.9% NaCl corresponds with a dose of approximately 40 mL/kg of NaCl solution, which according to previous reports is considered hypertonic when given in a single injection [46]. Hypertonic saline solutions were previously shown to improve the DAI in DSS-treated mice, which was associated with reduced inflammation on day 7 [46]. In our hands, fluid supplementation had no effects on any parameter associated with inflammation on day 7 suggesting that epithelial restitution occurred during the phase of recovery. The process of intestinal wound healing occurs through sequential steps that involve IEC migration, proliferation, and differentiation [27, 47]. Each one of these steps depends on the coordinated action of diverse signaling pathways that control epithelial homeostasis throughout the crypt axis, including the Wnt/β-catenin, EGFR, and TGF-β signaling pathways [25]. In the initial phases, IECs adjacent to the edges of the wound migrate over the wound bed to restore the epithelial monolayer, while stem and transit amplifying cells proliferate to restore the pool of IECs [47, 48]. IEC migration is enhanced by FGFs in a TGF-β-dependent manner [49], as well as by activation of MAPK such as ERK1/2 [50, 51]. However, TGF-β is a well-known repressor of mitogenic activity in the stem cell niche, whereas activation of ERK1/2 also promotes proliferation [25, 36]. In our model, fluid supplementation increased ERK1/2 activation and reduced TGF-β expression during the acute phase of colitis. This shift in signaling was associated with a hyperproliferative phenotype in crypts that was accompanied by upregulation of the Wnt target gene Axin-2, suggesting the involvement of this signaling pathway in the fluid-induced response [52]. Of note, activation of the Wnt signaling pathway is essential to overcome the inhibitory effects of proinflammatory cytokines in IEC proliferation during inflammation [53].

In later phases of the epithelial healing process, activation of TGF-β plays pivotal roles in the regeneration of crypt structure and in IEC differentiation [36–38]. Interestingly, we observed an increased expression of TGF-β in fluid-supplemented mice in the latter phases of epithelial recovery (day 12), suggesting that regenerative phases also occurred earlier in this experimental group. Final stages of epithelial wound repair involve the differentiation of dividing IECs into absorptive and secretory lineages [26, 27]. Our data show that accelerated recovery in the acute phase of colitis (day 7) was accompanied by an earlier differentiation of hyperproliferative IECs into secretory goblet cells on day 12. Moreover, fluid supplementation upregulated the expression of *Anpep*, a marker for absorptive enterocytes, further suggesting that the whole process of epithelial recovery after injury was enhanced by this intervention. Although no significant changes were observed in a functional test for epithelial permeability, earlier wound repair led to diminished inflammation scores in fluid-supplemented mice on day 12. This finding could be explained by a decreased exposure of the mucosa to the luminal content in these mice, as we observed a significant reduction in the areas of epithelial erosion and ulceration in fluid-supplemented mice. Since microbial populations are known to be causative or exacerbating factors of colitis in spontaneous IBD models, earlier reepithelization may contribute to the subsequent reduction of inflammation [54, 55].

Altogether, the results of our study argue against routine fluid supplementation in mouse models of acute colitis. In the absence of apparent dehydration, fluid IP administration neither improved animal stress, as defined by behavioral responses, nor inflammation, as determined by systemic and tissue-specific parameters. Instead, we witnessed a profoundly altered profile of epithelial wound repair and crypt proliferation, subsequently leading to an earlier recovery of the epithelial lining. These observations have important implications in experimental design, as researchers considering this welfare intervention must be aware of the changes in pathway activation, gene expression, and functional responses it causes. Furthermore, our findings support the use of fluid therapy in IBD patients, not only because preserving hydration status is essential to maintain a good general body condition, but because it may improve epithelial healing during disease flares.

## Supporting information

S1 Fig Fluid supplementation does not change hydration status or anxiety-like behavior in the open field. **A)** Water consumption per day and mouse during DSS administration (n = 3 different cages per group). ANOVA detected a significant “Time” effect (*P*<0.0001) but no differences between for “Fluid” factor. **B)** Fecal blood (n = 16–17 until day 7, n = 9 from days 7–12; *P<0.05 and **P<0.01, as determined by two-way ANOVA). ANOVA detected “Fluid” as a significant source of variation (*P*<0.01). DSS administration days are represented with a continuous line; fluid administration days are represented with a dashed line. **C)** Stool consistency (n = 16–17 until day 7, n = 9 from days 7–12; *P<0.05, **P<0.01 and ***P<0.001, as determined by two-way ANOVA). ANOVA detected “Fluid” as a significant source of variation (*P*<0.0001). DSS administration days are represented with a continuous line; fluid administration days are represented with a dashed line. **D)** Plasma total protein concentration (n = 8–9; **P<0.01, as determined by two-way ANOVA). **C)** Locomotive activity of mice in the center of the open field (n = 11–12; two-way ANOVA). **D)** Locomotive activity in the periphery of the open field (n = 11–12; two-way ANOVA). Our values differ from other studies because of the settings used in the open field determinations (center area is larger than peripheral).
(TIF)

S2 Fig Expression of proinflammatory cytokines in the colon is unmodified by fluid supplementation. **A)** IL-1β mRNA expression levels (n = 3–6; two-way ANOVA). ANOVA detected a significant “Time” effect (*P*<0.0001) but no differences for “Fluid” factor. **B)** TNF mRNA expression levels (n = 4–6; two-way ANOVA). ANOVA detected a significant “Time” effect (*P*<0.0001) but no differences for “Fluid” factor.
(TIF)

S3 Fig Fluid supplementation upregulates proliferative gene transcripts. **A)** Ki67 mRNA expression levels (n = 4–5; *P<0.05 and ** *P*<0.01 for fluid vs control on days 7 and 12, as determined by two-way ANOVA). **B)** Axin-2 mRNA expression levels (n = 4–5; **P<0.01 for fluid vs control on day 12, as determined by two-way ANOVA). ANOVA detected significant “Time” (*P*<0.01) and “Fluid” (*P*<0.05) overall effects.
(TIF)

S1 Table List of primers used in this study. (DOCX)

S2 Table Raw data underlying the findings described. (XLSX)
