# Supplementary figures and images for: Fluid supplementation accelerates epithelial repair during chemical colitis
Source: PLoS One. 2019 Apr 19;14(4):e0215387. doi: 10.1371/journal.pone.0215387 (PMC6474653; doi:10.1371/journal.pone.0215387)

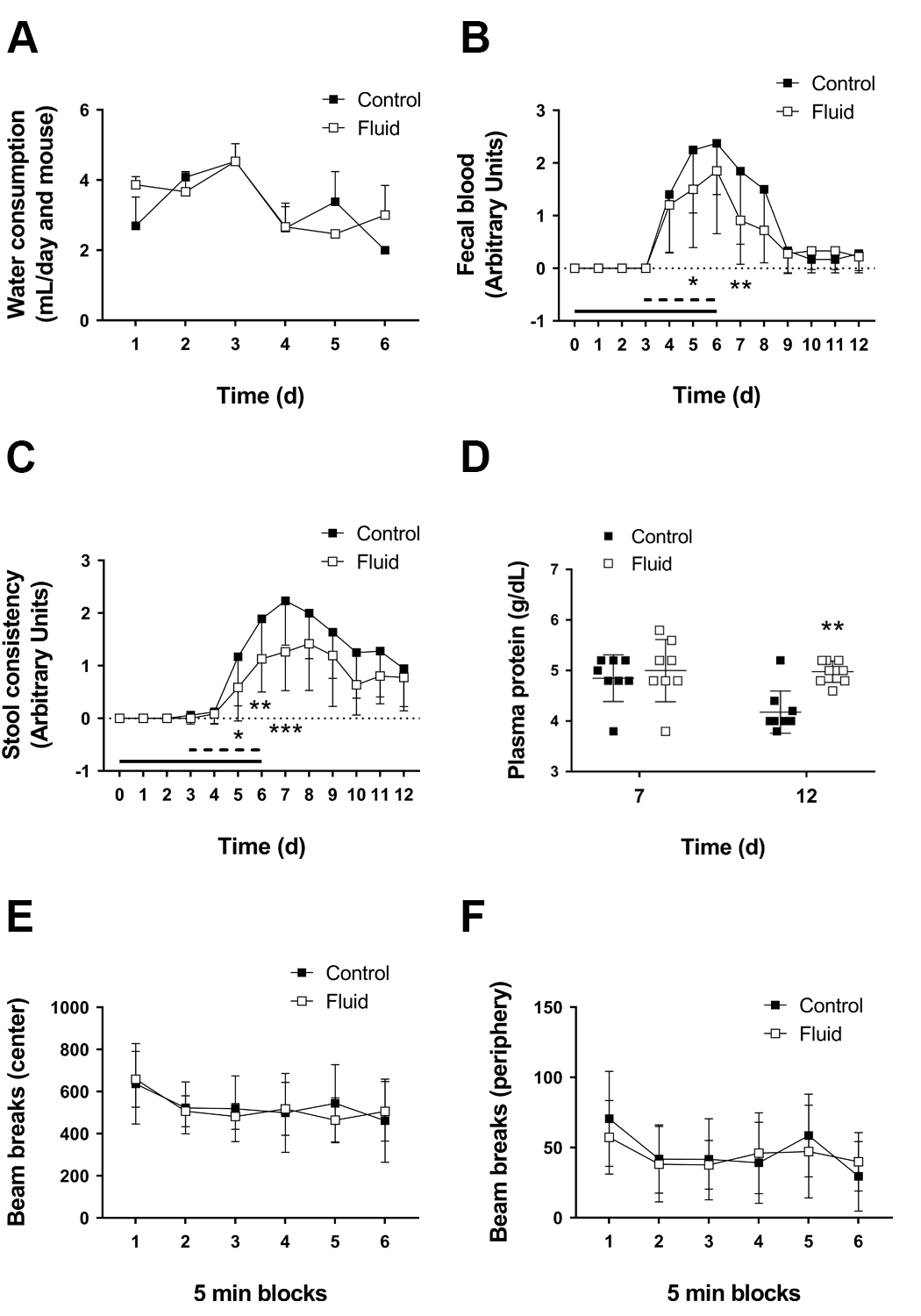

Supplement: S1 Fig — A) Water consumption per day and mouse during DSS administration (n = 3 different cages per group). ANOVA detected a significant “Time” effect (P<0.0001) but no differences between for “Fluid” factor. B) Fecal blood (n = 16–17 until day 7, n = 9 from days 7–12; *P<0.05 and **P<0.01, as determined by two-way ANOVA). ANOVA detected “Fluid” as a significant source of variation (P<0.01). DSS administration days are represented with a continuous line; fluid administration days are represented with a dashed line. C) Stool consistency (n = 16–17 until day 7, n = 9 from days 7–12; *P<0.05, **P<0.01 and ***P<0.001, as determined by two-way ANOVA). ANOVA detected “Fluid” as a significant source of variation (P<0.0001). DSS administration days are represented with a continuous line; fluid administration days are represented with a dashed line. D) Plasma total protein concentration (n = 8–9; **P<0.01, as determined by two-way ANOVA). C) Locomotive activity of mice in the center of the open field (n = 11–12; two-way ANOVA). D) Locomotive activity in the periphery of the open field (n = 11–12; two-way ANOVA). Our values differ from other studies because of the settings used in the open field determinations (center area is larger than peripheral). (TIF) [file pone.0215387.s001.tif]

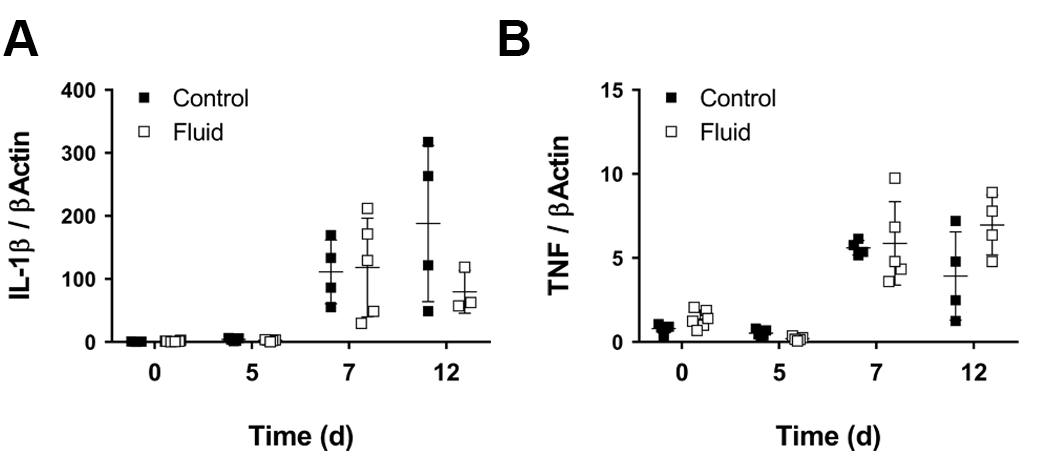

Supplement: S2 Fig — A) IL-1β mRNA expression levels (n = 3–6; two-way ANOVA). ANOVA detected a significant “Time” effect (P<0.0001) but no differences for “Fluid” factor. B) TNF mRNA expression levels (n = 4–6; two-way ANOVA). ANOVA detected a significant “Time” effect (P<0.0001) but no differences for “Fluid” factor. (TIF) [file pone.0215387.s002.tif]

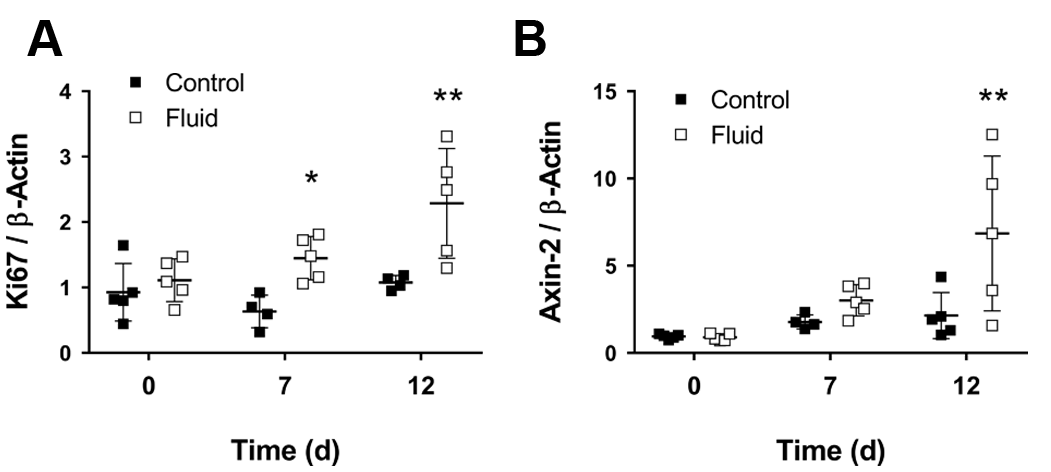

Supplement: S3 Fig — A) Ki67 mRNA expression levels (n = 4–5; *P<0.05 and ** P<0.01 for fluid vs control on days 7 and 12, as determined by two-way ANOVA). B) Axin-2 mRNA expression levels (n = 4–5; **P<0.01 for fluid vs control on day 12, as determined by two-way ANOVA). ANOVA detected significant “Time” (P<0.01) and “Fluid” (P<0.05) overall effects. (TIF) [file pone.0215387.s003.tif]
