# Supplementary material for: Fluid supplementation accelerates epithelial repair during chemical colitis
Source: PLoS One. 2019 Apr 19;14(4):e0215387. doi: 10.1371/journal.pone.0215387 (PMC6474653; doi:10.1371/journal.pone.0215387)
Supplement: S1 Table — (DOCX) [file pone.0215387.s004.docx]

| **Gene** | **Sense primer** | **Antisense primer** | **Reference** |
| --- | --- | --- | --- |
| Anpep | CCTGTAGCAAAGATGTGTGGATT | GGATGAGATTGGCAAAGGAGAAA | [56] |
| Axin-2 | GAGAGTGAGCGGCAGAGC | CGGCTGACTCGTTCTCCT | [57] |
| β-Actin | TGACAGGATGCAGAAGGAGA | CGCTCAGGAGGAGCAATG | [58] |
| ChgA | CGATCCAGAAAGATGATGGTC | CGGAAGCCTCTGTCTTTCC | [59] |
| EREG | TTGTGCTGATAACTGCCTGTAGAA | CACCGAGAAAGAAGGATGGAGAC | [33] |
| FGF-2 | CAACCGGTACCTTGCTATGA | TCCGTGACCGGTAAGTATTG | [60] |
| IL-1β | AGTTGACGGACCCCAAAAG | AGCTGGATGCTCTCATCAGG | [61] |
| Ki67 | GATGGAAGCATTGTGAGAACCA | CCTGCTCTTCCACAGATTCAAG | [56] |
| Muc2 | GTGGATGTGTGGGACCTGA | TTGCAGTCAAACTCAAAGTGC | [62] |
| TGF-β1 | TGGAGCAACATGTGGAACTC | CAGCAGCCGGTTACCAAG | NM_011577.2 |
| TNF | TCTTCTCATTCCTGCTTGTGG | GGTCTGGGCCATAGAACTGA | [63] |
